# A comparative metabolomics analysis of domestic yak (*Bos grunniens*) milk with human breast milk

**DOI:** 10.3389/fvets.2023.1207950

**Published:** 2023-09-29

**Authors:** Wenhao Li, Weike Zeng, Yanping Zhang, Zhijie Ma, Xingyan Fang, Yingcang Han, Yonggang Sun, Xiayang Jin, Liuyin Ma

**Affiliations:** ^1^Institute of Animal Husbandry and Veterinary Science, Qinghai University, Xining, China; ^2^Center for Genomics, Fujian Provincial Key Laboratory of Haixia Applied Plant Systems Biology, Fujian Agriculture and Forestry University, Fuzhou, China; ^3^College of Life Sciences, Fujian Agriculture and Forestry University, Fuzhou, China

**Keywords:** colostrum milk, mature milk, yak, human breast, comparative metabolomics, hypoxic stress tolerance, GC–MS

## Abstract

Yaks are tough animals living in Tibet’s hypoxic stress environment. However, the metabolite composition of yak milk and its role in hypoxic stress tolerance remains largely unexplored. The similarities and differences between yak and human milk in hypoxic stress tolerance are also unclear. This study explored yak colostrum (YC) and yak mature milk (YMM) using GC–MS, and 354 metabolites were identified in yak milk. A comparative metabolomic analysis of yak and human milk metabolites showed that over 70% of metabolites were species-specific. Yak milk relies mainly on essential amino acids- arginine and essential branched-chain amino acids (BCAAs): L-isoleucine, L-leucine, and L-valine tolerate hypoxic stress. To slow hypoxic stress, human breast milk relies primarily on the neuroprotective effects of non-essential amino acids or derivates, such as citrulline, sarcosine, and creatine. In addition, metabolites related to hypoxic stress were significantly enriched in YC than in YMM. These results reveal the unique metabolite composition of yak and human milk and provide practical information for applying yak and human milk to hypoxic stress tolerance.

## 1. Introduction

Domestic yaks (*Bos grunniens*) are tough animals native to Tibet in alpine and subalpine regions at a latitude above 3,000 m with extreme cold and semi-humid climate (1). Up to 2019, more than 90% (~16 million) of the world’s yaks are distributed in western China’s Qinghai-Tibet Plateau. In 2020, the output value of China’s yak industry was around 6 billion dollars. Domestic yaks offer significant economic benefits to the Tibetans, and it has been used for farming and threshing, fur, meat, and high-quality milk (2). Yak milk and dairy products such as butter and cheese give Tibetans an essential source of vitamins and nutrients (1). Yak milk also plays a vital role in health protection. Hydrolysate of yak milk casein attenuates superoxide, DPPH, and hydrogen peroxide free radicals. It increases anti-inflammatory activities by preventing the secretion of pro-inflammatory cytokines-interleukins (IL-6, IL-1ß) and tumor necrosis factor-alpha (TNF-a) (3). Feeding yak milk to rats can stimulate the production of plasma immunoglobulin M (IgM) and enhance the immunity of rats (4). Yak milk can also improve intestinal microbes and prevent the growth of cancer cells (5, 6). Therefore, yak milk is vital to Tibetans’ health protection, economic income, and daily life. Unlike the minimal changes of bovine milk due to year-round breeding, the composition of yak milk is dynamic between warm and cold seasons as yaks show a robust seasonal breeding pattern (1). However, yak milk is still higher in fat (yak vs. bovine milk: 5.5–7.5% vs. 2.4–5.5%), lactose (yak vs. bovine milk: 4.0–5.9% vs. 3.8–5.3%), and protein (yak vs. bovine milk: 4.0–5.5% vs. 2.3–4.4%) than bovine milk (1). It is also reported that the protein content of yak milk is ~40–60% higher than that of native bovine milk (7). Thus, yak milk is highly nutritious and known as a naturally concentrated milk (1). Recently, yak milk has attracted much attention due to its unique characteristics mentioned above (1), and it is an urgent need to expand its production capacity.

The health and survival of newborn yak calves are the basis for expanding domestic yak populations and increasing yak milk production. Newborn calves are vulnerable to diseases, and colostrum is vital in feeding and managing newborn bovine calves (8, 9). Colostrum management is still considered the most critical factor in preventing bovine calf morbidity and mortality (9). Colostrum is the primary source of nutrients for newborn calves and the only source of passive immunity for calves (8, 9). During pregnancy, due to the barrier effect of the placenta, the calf cannot directly obtain immunoglobulin, and the active immunity of the newborn calf has not yet developed (8, 9). Therefore, passive immunity can only be acquired by ingesting colostrum against the unfavorable external environment and harmful bacteria infection (8, 9). More than 15.6% of newborn calves have serum IgG concentrations below 10 g/L at 24 to 48 h, and calves below this threshold are at a higher risk of death, resulting in a phenomenon known as the failure of passive transfer (FPT) (8, 10). Overall, colostrum benefits subsequent calf and adult development (8, 11). In addition, hypoxic stress is one of the significant environmental stresses for domestic animals living in high-altitude regions (12). However, the roles of colostrum on hypoxic stress tolerance and the development of yak calves are mainly unexplored.

Recently, it has been reported that there are significant differences in the content of triglycerides (13) and protein (14), the composition of fatty acids at different milk lactation stages (15, 16). It has been reported that ruminant milk provides a variety of essential nutrients that affect the contents of various immune factors of mammals (4). The composition of donkey milk at different lactation stages contains different content of long-chain fatty acids, which affect the immunological development of infants (17). However, the changes in the composition between yak colostrum (YC) and yak mature milk (YMM) have yet to be reported.

Metabolomics analysis involves the systematic study of low molecular weight (< 1kda) endogenous and exogenous metabolites (such as lipids, amino acids, and organic acids), which represent the genetic and environmental intersection of cell functions and effects (18, 19). This method has recently been recognized as a promising tool for studying the nutritional quality of milk (20). However, previous studies on milk metabolomics focused on different species of mammals (21) or other physiological states of mammals (22). Recently, metabolomics has been used to study donkey milk between colostrum and mature milk (16, 23). These pioneer reports fully illustrate the reliability of metabolomics in milk research. However, the changes in the metabolite composition between YC and YMM remain unexplored.

In this study, the metabolites of yak colostrum (YC) and yak mature milk (YMM) were investigated by gas chromatography–mass spectrometry (GC–MS). Comparative metabolomic analysis identified distinct metabolites and explored the mechanisms underlying compositional changes between YC and YMM. The comparative metabolomics between yak and human breast milk was also analyzed. Common metabolites between yak and human breast milk were also analyzed in detail. This study provides practical information on the nutritional components of yak milk and lays a foundation for the development and research of yak milk products.

## 2. Materials and methods

### 2.1. Milk sample collection

The yak milk samples were collected from the “Yushu Yak” at the Zangbala Farm (E89°27′ ~ 97°39′; N31°45′ ~ 36°10′) in Yushu, Qinghai Province, China. Ninety cows with similar nutritional levels were selected as the experimental group. Six yak YC or YMM samples were randomly chosen for metabolomics analysis, respectively. These yaks were all around six years old and under similar physical conditions. The milk sample was frozen to liquid nitrogen and stored at −80°C until analysis.

The human breast milk data was from a previous publication and the human breast milk sampling method is described again (24). Briefly: 60 first-born mothers in the same hospital participated; 30 volunteered to provide Human Colostrum (HC, collected at 1–5 days postpartum). Human Mature Milk (HMM) was provided by 30 volunteers (collected at 30–40 days postpartum). Six biological replicates were sampled for HC and HMM, respectively. Each biological replicate was collected by randomly mixing 5 milk samples to reduce the difference due to dietary and other habits. The milk sample was frozen to liquid nitrogen and stored at −80°C until analysis.

### 2.2. Chemicals and quality control samples

The chemical reagents and the catalog numbers were identical to the previous study (23). The methanol, H_2_O, Pyridine, N-hexane, O-methyl hydroxylamine hydrochloride (97%), and BSTFA+1%TMCS were all from CNW technologies. The acetonitrile and 2-chloro-l-phenylalanine were separately provided by Fisher and Shanghai HC Biotech Co. The eleven fatty acid methyl esters were supported by Larodan, Nc-chek, DR. The quality control (QC) samples were prepared by equally mixing the metabolite extracts from 12 samples, including six Yak colostrum and six mature milk samples. The metabolites were identified based on three identical QC samples to increase the reliability of experiments.

### 2.3. Sample extracts

The sample extracts were prepared following the standard protocol for GC–MS metabolomics analysis (25). Briefly, 80 μL of each sample was mixed with 20 μL of internal standard (2-chloro-l-phenylalanine in methanol, 0.3 mg/mL) and then extracted with 240 μL of extraction mixture (methanol: acetonitrile 2:1 vol/vol). The samples were then vortexed for 30 s and treated with an ultra-sound for 10 min on ice. Samples were then incubated on ice for 30 min at −20°C, followed by centrifuging at 13000 rpm for 10 min at 4°C. The supernatant (150 μL) was transferred into a new glass derivatization vial, and the quality control samples were prepared with identical procedures. The samples were evaporated by using a freeze-concentrated centrifugal dryer and 80 μL of methoxamine hydrochloride pyridine solution (15 mg/mL) to the glass derivatization vial to perform the oximation reaction in a shaking incubator at 37°C for 90 min. After adding 80 μL of BSTFA (with 1% TMCS) and 20 μL of n-hexane into the vial, 10 μL of 11 internal standards (C8/C9/C10/C12/C14/C16, 0.16 mg/mL; C18/C20/C22/C24/C26, 0.08 mg/mL, chloroform configuration) were added into samples to react at 70°C for 60 min. After the reaction, the samples were placed at room temperature for 30 min for GC–MS metabolomics analysis.

### 2.4. GC–MS analysis

The derivatized samples were analyzed on an Agilent 7890B gas chromatography system coupled to an Agilent 5977A MSD system (Agilent Technologies Inc., CA, USA) with standard protocol (25). A DB-5MS fused-silica capillary column (30 m × 0.25 mm × 0.25 μm, Agilent J & W Scientific, Folsom, CA, USA) was utilized to separate the derivatives. Helium (>99.999%) was used as the carrier gas at a constant flow rate of 1 mL/min through the column. The injector temperature was maintained at 260°C. The injection volume was 1 μL by splitless mode. The initial oven temperature was 60°C, ramped to 125°C at a rate of 8°C/min, to 210°C at a rate of 5°C/min, to 270°C at a rate of 10°C/min, to 305°C at a rate of 20°C/min, and finally held at 305°C for 5 min. The MS quadrupole and ion source (electron impact) temperature was set to 230°C, and the quadrupole temperature was set to 150°C. The collision energy was 70 eV. Mass spectrometric data were acquired in a full-scan mode (m/z 50–500). The QCs were injected at regular intervals (every six samples) throughout the analytical run to provide data from which repeatability could be assessed.

### 2.5. Data processing

The GC/MS raw data were processed by AnalysisBaseFileConverter software and then imported into MS-DIAL software for further processing to get the original data matrix (26). The Untarget database of GC–MS from Lumingbio was used for identifying metabolite. After removing the data noise of the data matrix, the normalization of each sample and log conversion was performed to get the raw, clean matrix.

### 2.6. Bioinformatics analysis

The raw data clean matrix was then imported into the R ropls package for Principal component analysis (PCA), (orthogonal) partial least-squares-discriminant analysis (O)PLS-DA (27). Correlation analysis uses the Pearson correlation coefficient and the structural similarity of metabolites to calculate the correlation between two metabolites and then visualizes it through the Cytoscape 3.8.2 software (28). In addition, the untarget database of GC–MS from Lumingbio and the KEGG database (https://www.kegg.jp/kegg/) was used for qualitative and metabolic pathway analysis.

### 2.7. Comparative analysis of different milk metabolite

The human breast milk metabolomics data were obtained from the previous publication (24). Common metabolites were identified by identical PubChem CID in different milk. After this, calculate each species’ Fold Change value (FC) between mature milk and colostrum. Differential common metabolites identified by |FC_(species 1)_ – FC_(species 2)_| > 1.

## 3. Results

### 3.1. Statistical comparison of metabolites between YC and YMM

According to the GC–MS data, a total of 354 metabolites were identified in Yak milk (Supplementary Table S1), and the signal intensity (peak area) of all peaks was normalized according to the internal standard with RSD (relative standard deviation) (ALL) < 0.3 after the screening. The missing values in the raw data were replaced by zero. In particular, the YC (Yak colostrum) and YMM (mature yak milk) groups were separated from each other by principal component analysis using MS (Mass Spectrometry) raw data (Supplementary Figure S1A). Next, orthogonal partial least squares-discriminant analysis (OPLS-DA) was used to filter out the noise that has nothing to do with classification information to improve the analytical ability and effectiveness of the mode (Supplementary Figure S1B). The model parameters were R2Y = 0.998 and Q2Y = 0.972, indicating that the model could successfully classify samples. The 7-fold cross-validation and 200 response permutation testing (RPT) methods were used to further examine the model’s quality. The results unveiled that the model was stable and the risk of overfitting was lower (Supplementary Figure S1C). Accordingly, a loading graph was constructed based on OPLS-DA (Supplementary Figure S1D) to better visualize the important variables far from the origin and their contribution to the difference between the YC and YMM.

### 3.2. Identification of significantly different metabolites between YC and YMM

**Figure 1:**
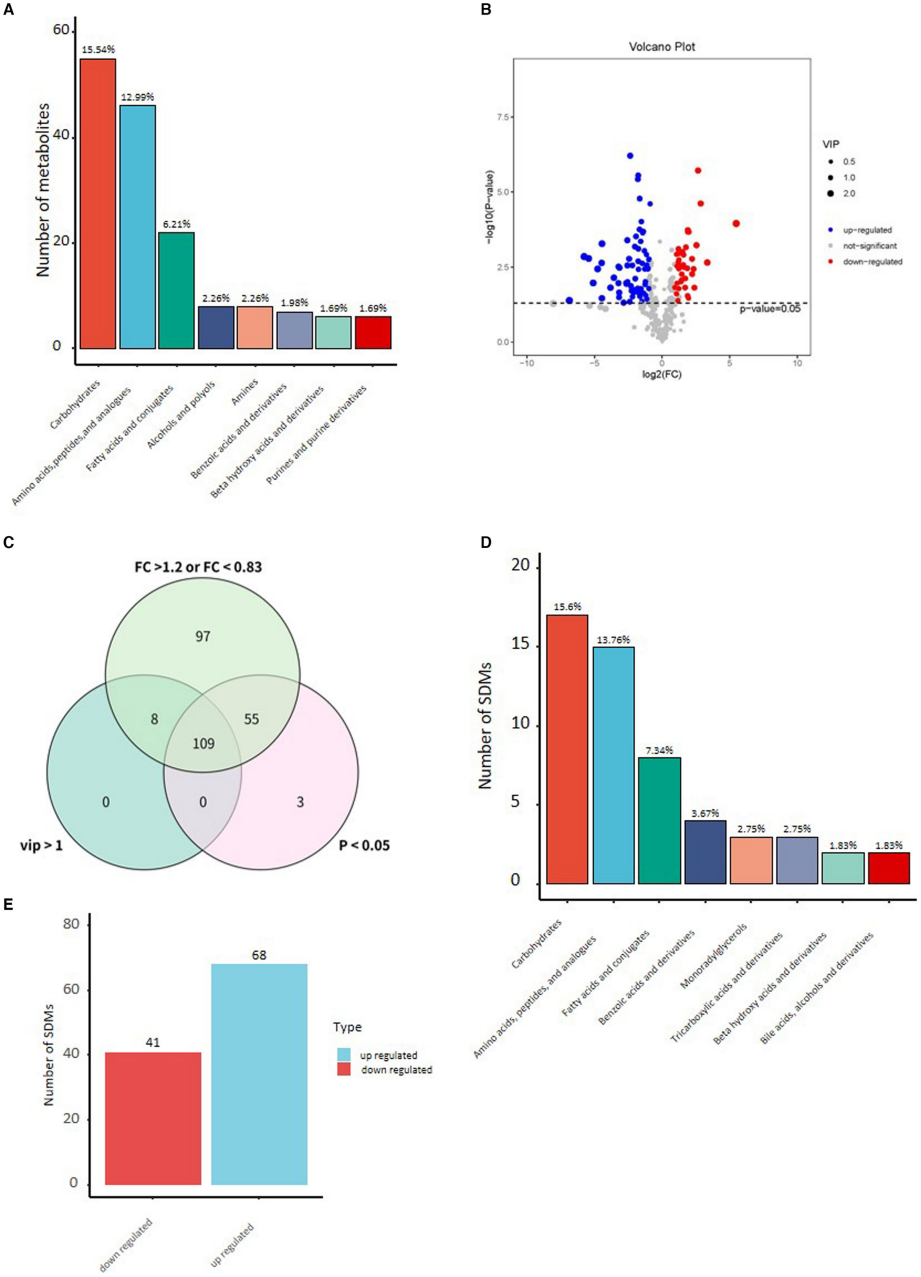
**(A)** Histogram of the top 8 categories in the classification of stage milk identified metabolites and their percentages to the total number of metabolites. **(B)** Volcano plot of differential metabolites in yak milk (YC vs. YMM). (Student’s t-test, *p* < 0.05, FC >1.2 or FC < 0.83). **(C)** Venn diagram of different criteria to screen for significant differential metabolites. **(D)** Histogram of top 8 type significant differential metabolites (Student’s *t*-test: *p* < 0.05, FC >1.2 or FC < 0.83, VIP > 1). **(E)** Histogram of significant up or down-regulated metabolites in YC compared to YMM. YC: yak colostrum; YMM: yak mature milk; FC: Fold Change; VIP: variable importance in the projection.

**Figure 2:**
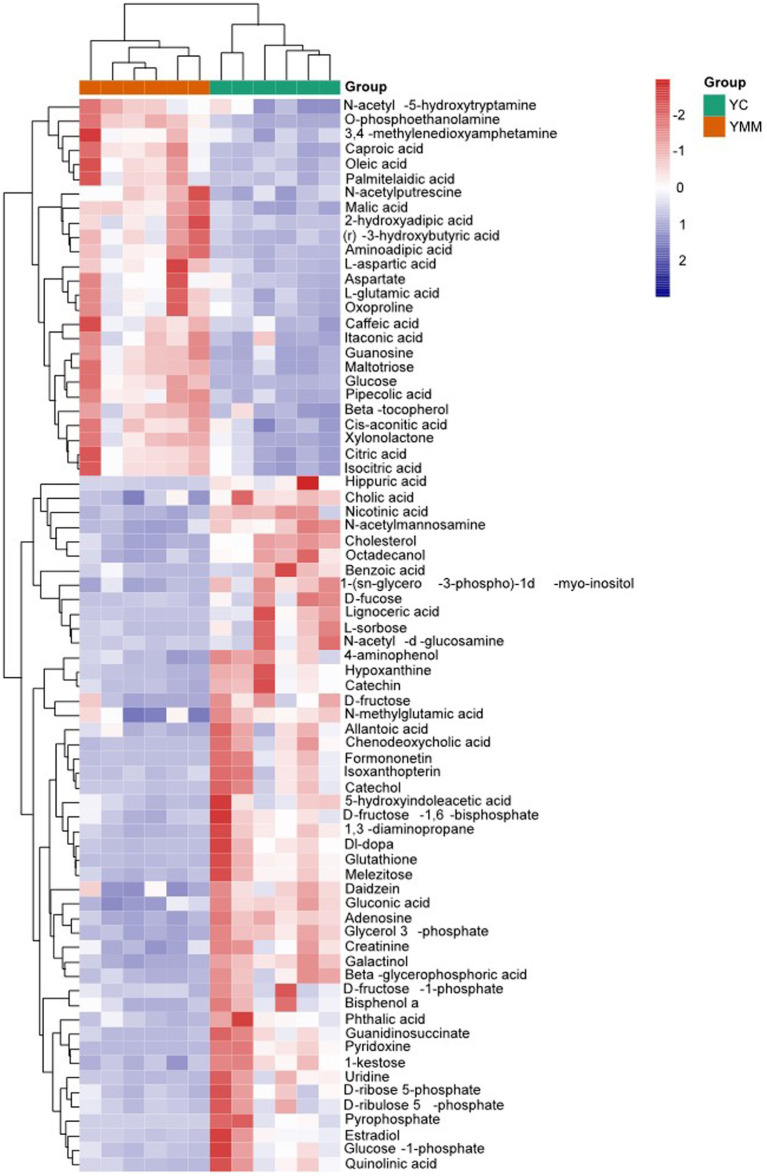
Heatmap of known significantly different metabolite (KSDM). SDMs: significantly different metabolites; KSDM: SDMs can be annotated to the KEGG pathway.

By comparing with the Untarget database of GC–MS from Shanghai luming biological technology co. Ltd., the three most abundant metabolites of Yak milk were carbohydrates and carbohydrate conjugates (15.54%), amino acids (12.99%), fatty acids, and conjugates (6.21%), which accounted for 38.89% of all 354 identified metabolites (Figure 1A). To understand the changes of metabolites in Yak milk between YC and YMM, different metabolites have been identified between the YC and YMM. As shown in the Volcano map (Figure 1B), a total of 164 different metabolites (FC > 1.2, or FC < 0.83, student *t-*test: *p* < 0.05) were identified, and 109 of them were selected as significantly different metabolites (SDM) between the YC and YMM groups with an additional criterion: the variable importance in the projection (VIP) values exceeding 1.0 (Figure 1C, Supplementary Tables S2–S4). Interestingly, carbohydrates and carbohydrate conjugates (4), amino acids (15), fatty acids and conjugates (8) were consistently the most abundant SDMs (Figure 1D). However, the percentage of all three groups of metabolites was increased in SDMs compared to total Yak milk metabolites, respectively. Significantly, the rate of fatty acids and conjugates (a 1.13% increase, SDM vs. total Yak metabolites: 7.34% vs. 6.21%) increased in SDMs compared with that from whole Yak milk (Supplementary Figure S2). Metabolites related to tricarboxylic acids and derivatives, monoradylglycerols and benzoic acids and derivatives were also among SDMs (Supplementary Table S2). In addition, the number of up-regulated metabolites was higher than (68 vs. 41) from down-regulated ones in YC compared to YMM (Figure 1E). 74 SDMs can be annotated to the KEGG pathway. They were called known significantly different metabolites (KSDM) (Figure 2). These results indicated that the metabolite composition was changed considerably between YC and YMM.

### 3.3. Correlation network analysis of the SDMs

**Figure 3:**
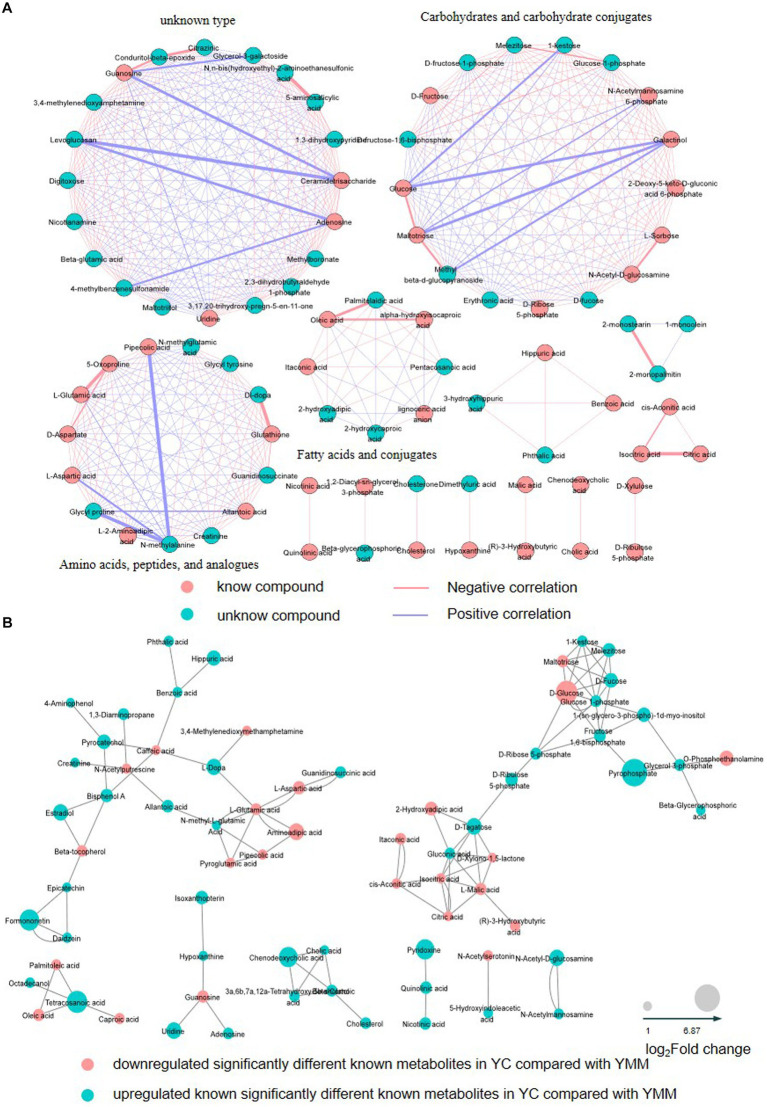
Correlation network graph of SDMs and KSDMs. **(A)** The Correlation network graph based on changes in relative content and the type of SDMs. Red lines show a positive correlation; blue lines indicate a negative correlation; pink circles show a known metabolite; blue circles show an unknown metabolite. **(B)** The correlation network graph is based on known significantly different metabolites’ (KSDM) chemical structure and physiological function. The red nodes represent that KSDMs are significantly down-regulated (YC vs. YMM), and the blue nodes represent that KSDMs are significantly up-regulated (YC vs. YMM). The node size represents the multiple of the difference in metabolite content. YC: yak colostrum; YMM: yak mature milk; SDMs: significantly different metabolites; KSDM: SDMs can be annotated to the KEGG pathway.

To identify the relationship between different SDMs, the correlation between metabolites was calculated using the Pearson correlation coefficient and was then grouped according to the type of metabolite (Supplementary Table S3). Visualization with Cytoscape, there were 478 edges, with red lines showing a positive correlation, blue lines showing a negative correlation, pink circles showing a known metabolite, and blue circles showing an unknown metabolite (Figure 2). A total of 14 clusters could be classified according to the type of metabolites (Figure 2, Supplementary Table S5). These results revealed the association of metabolites within the group. Carbohydrates and their conjugates, amino acids, peptides, and analogs, and fatty acids and their conjugates were the three most closely related to each other (Figure 3A).

74 KSDMs are uploaded to the web-based MetaboAnalyst (https://www.metaboanalyst.ca) (29) for further analysis. Based on the metabolites’ chemical structure and physiological function, another correlation network analysis found that KSDM was highly correlated. These KSDMs were grouped into eight clusters, which revealed the association of KSDM between YC and YMM (Figure 3B). Amino acid metabolism with other metabolism and glycometabolism were the most closely related in the 8 clusters, and KSDM in glycometabolism had almost no association with other KSDM (Figure 3B). These results indicated that the regulatory network among KSDM was complex.

### 3.4. Key differential metabolic pathways between YC and YMM

**Figure 4:**
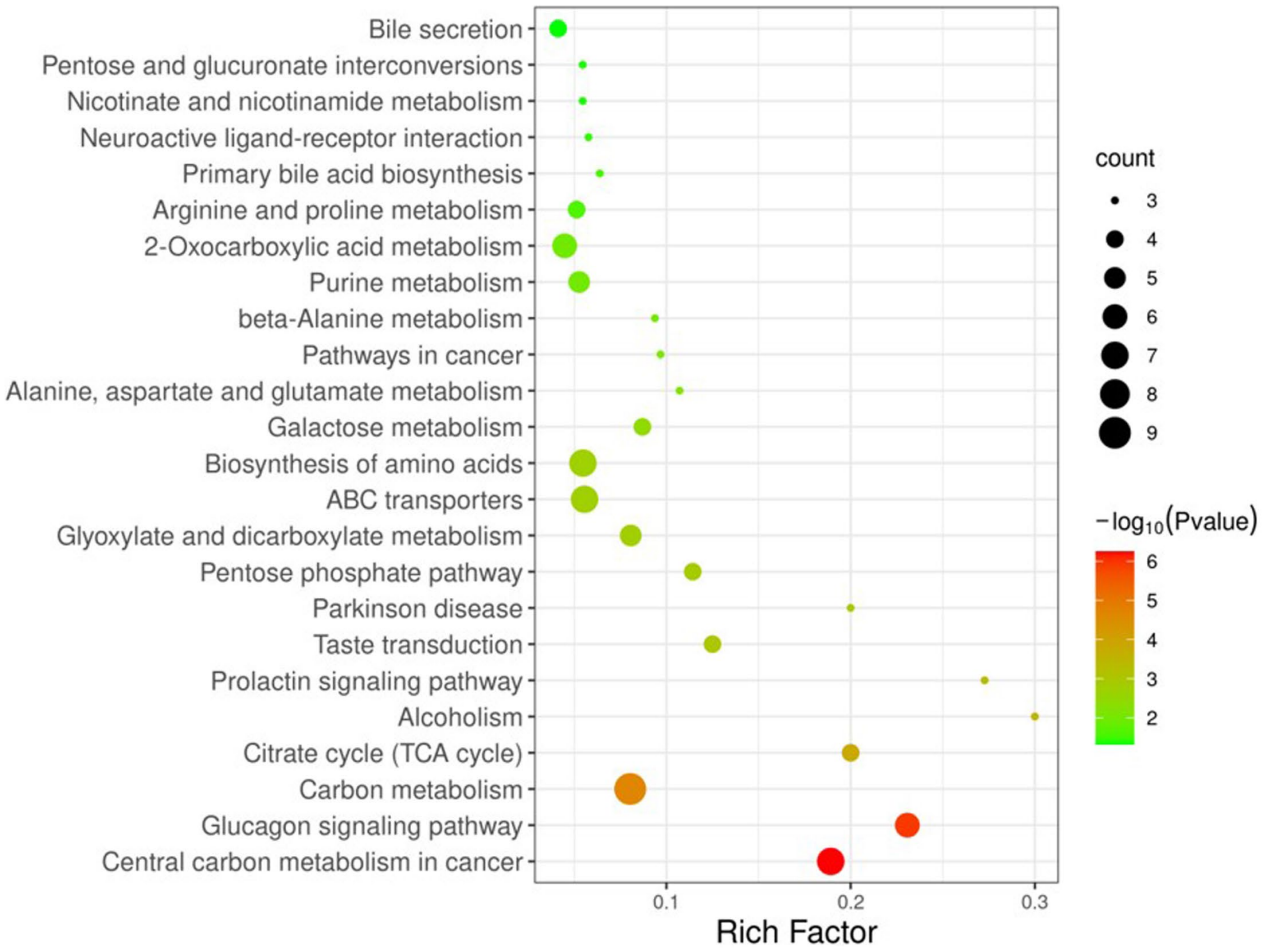
KEGG pathway enrichment map of known significantly different metabolite. KSDM: SDMs can be annotated to the KEGG pathway; Criteria: *p* < 0.05, The number of metabolites enriched in each KEGG pathway is more than two.

**Figure 5:**
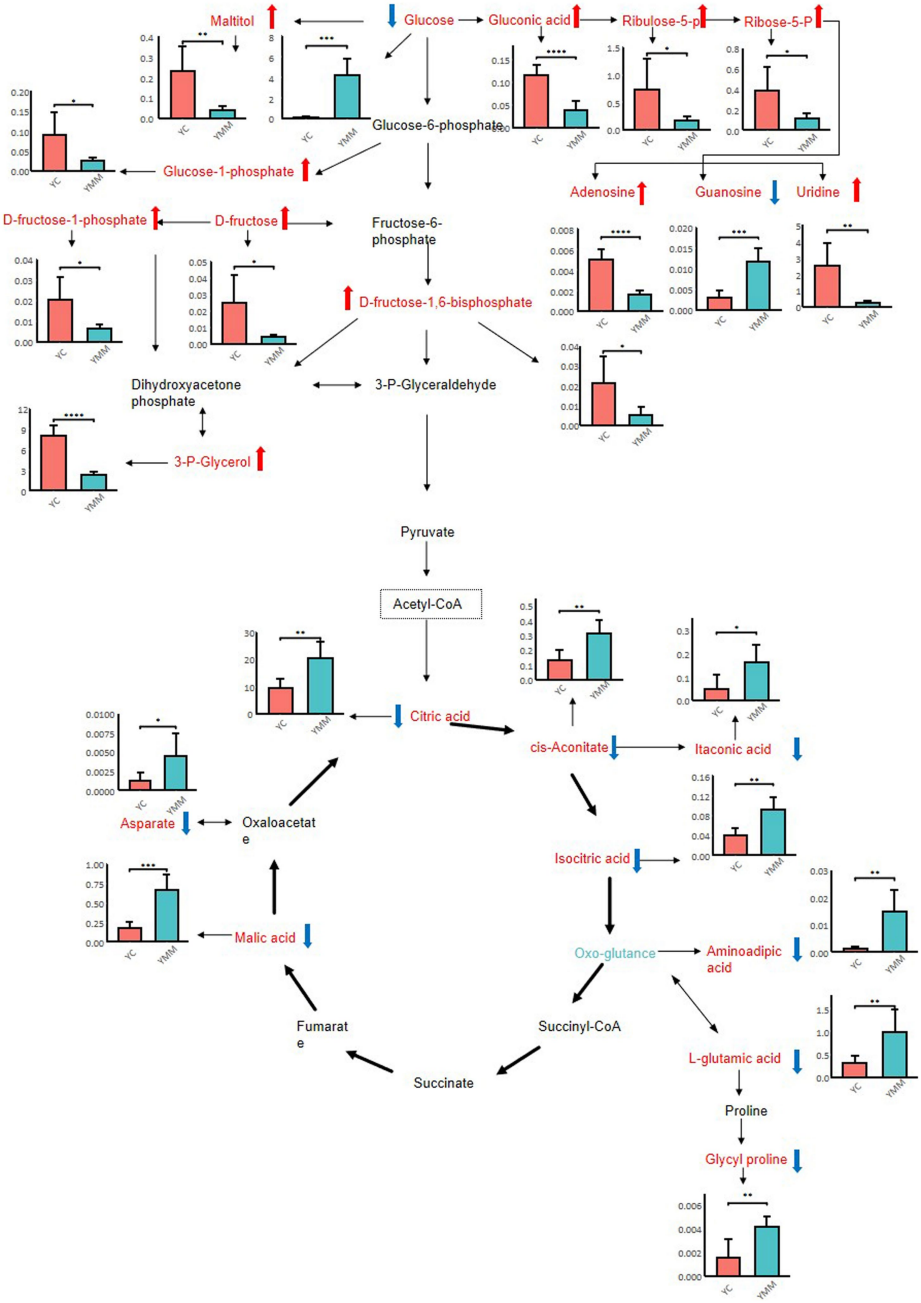
Hypothesized scheme pathway and related SDMs most related to metabolite changes of yak milk during lactation. Error bars of each bar plot represent the standard deviation of six biological replicates; student *t*-test: *p < 0.05; **p < 0.01; ***p < 0.001; ****p < 0.0001.

To better understand the changes in components between YC and YMM, 74 KSDMs were sent to the untargeted database of GC–MS from Lumingbio and KEGG databases. These metabolites were enriched into 116 different metabolic pathways. Based on the *p* value and the number of metabolites, 24 key pathways were screened out, which might correlate with the metabolism composition dynamics between YC and YMM (Figure 4). The top 15 metabolic pathways of KSDMs are central carbon metabolism in cancer, glucagon signaling pathway, carbon metabolism, citrate cycle (TCA cycle), alcoholism, prolactin signaling pathway, taste transduction, parkinson disease, pentose phosphate pathway, glyoxylate and dicarboxylate metabolism, ABC transporters, biosynthesis of amino acids, galactose metabolism, alanine, aspartate and glutamate metabolism, pathways in cancer (Figure 4). Based on these critical metabolic pathways and other related SDMs, a detailed pathway map of metabolic changes between YC and YMM was constructed (Figure 5). Levels of glucose and TCA cycle metabolites such as citric acid, cis-aconitic acid, isocitrate and malic acid were lower in YC than in YMM (Figure 5). In contrast, metabolites related to the pentose phosphate pathway, including Ribulose-5-P and Ribose-5-P, were upregulated in YC compared to YMM (Figure 5). Overall, these results suggest that the metabolite composition of YC is different from that of YMM.

### 3.5. Comparative metabolomic analysis of yak and human breast milk

**Figure 6:**
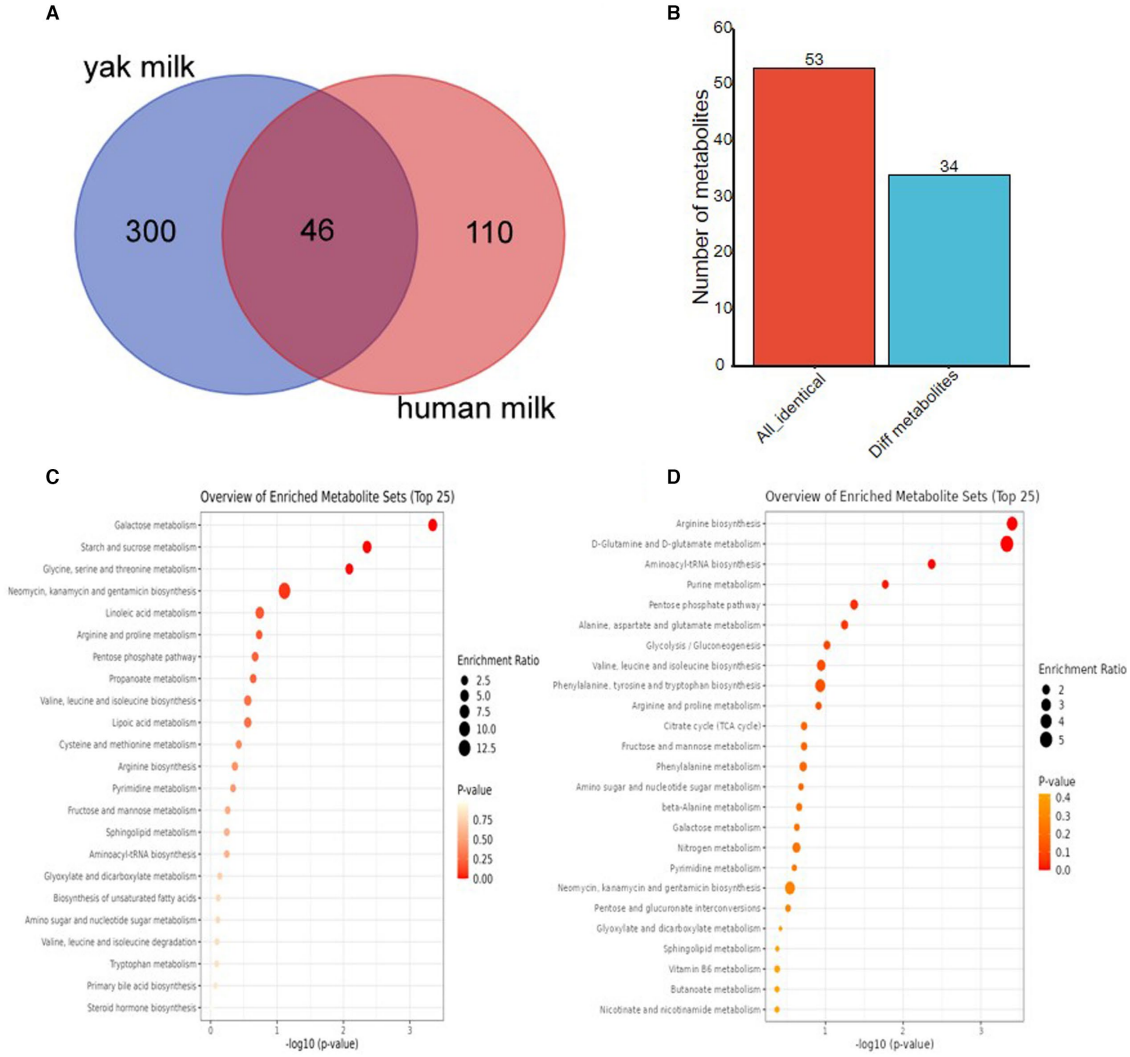
Comparative metabolomics analyses among yak milk and human breast milk. **(A)** Venn gram to represent the metabolites composition among milk from three species by PubChem CID. **(B)** Bar plot to characterize the metabolites with differentially expressed trends between colostrum and mature milk in yak and humans. **(C)** KEGG pathway analysis of human breast milk-specific metabolites. **(D)** KEGG pathway analysis of yak milk-specific metabolites. PubChem CID: PubChem Compound Identifier.

To further explore the metabolic composition of yak milk, comparative metabolomic studies were performed in yak and human breast milk, as colostrum and mature milk with GC–MS metabolomic data are available only for these two species. We first compared the metabolite composition of the two kinds of milk with the PubChem CID. Yak milk shared 46 common metabolites with human breast milk (Figure 6A, Supplementary Table S6). To further explore whether yak and human milk share similar trends between colostrum and mature milk, we compared changes in 53 metabolites (corresponding to 46 common metabolites) in colostrum and mature milk from the two animals. The fold change of each metabolite between colostrum and mature milk was calculated from yak and human milk, respectively. The identification criteria for differently expressed metabolites were: |fold change (HC/HMM)-fold change (YC/YMM)| >1. Interestingly, more than 75.47% (40/53) of common metabolites had similar expression patterns between yak and human milk (Figure 6B, Supplementary Table S7). Therefore, yak and human have similar metabolite composition trends between colostrum and mature milk. Although only 13 metabolites have different expression patterns between yak and human milk, four of them including uridine, glutaric acid, L-isoleucine, and malonic acid were reported to be involved in the hypoxic stress response (29–32). Specially, uridine was decreased in HC than HMM (FC = 0.54950, HC vs. HMM: 0.07914 vs. 0.14402), whereas it was upregulated in YC than YMM (FC = 9.35992, YC vs. YMM: 2.49364 vs. 0.26642, Supplementary Table S7). Similarly, glutaric acid was reduced in HC than HMM (FC = 0.00563, HC vs. HMM: 0.00300 vs. 0.05285) while slightly increased in YC than HMM (FC = 1.14554, YC vs. YMM: 0.00130 vs. 0.00113, Supplementary Table S7). Conversely, L-isoleucine, one of the essential branched-chain amino acids (BCAA) (33–35), was increased more than two-fold in HC than HMM (FC = 2.65825, HC vs. HMM: 0.01260 vs. 0.00474) and only slightly increased in YC than YMM (FC = 1.20487, YC vs. YMM: 0.04986 vs. 0.04138, Supplementary Table S7). However, the L-isoleucine was more abundant in yak milk than in human breast milk (YC vs. HC: 0.04986 vs. 0.01260, YMM vs. HMM: 0.04138 vs. 0.00474, Supplementary Table S7). Furthermore, malonic acid was increased in HC than HMM (FC = 1.88758, HC vs. HMM: 0.04935 vs. 0.2614) but reduced in YC than YMM (FC = 0.68250, YC vs. YMM: 0.00060 vs. 0.00088, Supplementary Table S7). Thus, the different expression patterns of metabolites during lactation between yak and human milk that might contribute to the hypoxic stress responses.

Although yak and human milk share 46 metabolites, approximately 86.71% of the metabolites (300/346) in yak milk (303 metabolites, corresponding to 300 identical metabolites) are specific to yak and not found in human milk, according to PubChem CID (Supplementary Table S8). Similarly, according to PubChem CID (Supplementary Table S9), about 70.51% of the metabolites (110/156) in human milk (111 metabolites, corresponding to 110 identical metabolites) are specific to humans and not similar in yak milk. Therefore, yak milk and human milk differ significantly in the composition of metabolites.

KEGG enrichment analysis was performed for yak-specific metabolites and human breast-specific metabolites using HMDB IDs to explore the functions of yak-specific metabolites further. Galactose metabolism, starch and sucrose metabolism, glycine, serine, and threonine metabolism, neomycin, kanamycin, and gentamicin biosynthesis, and Linoleic acid metabolism are the most significantly enriched pathways in the human breast-specific metabolites (Figure 6C). Especially, serine, a non-essential amino acid (NEAA), was only presented in human breast milk but not in yak milk (Supplementary Tables S8, S9). In addition, the non-essential amino acids or derivates: citrulline, sarcosine, and creatine were only presented in human breast milk but not in yak milk (Supplementary Tables S8, S9). In contrast, yak-specific metabolites were enriched in arginine biosynthesis, D-glutamine, and D-glutamate metabolism, aminoacyl-tRNA biosynthesis, purine metabolism, and pentose phosphate pathways (Figure 6D). Interestingly, two other BCAAs: L-leucine and L-valine, were only presented in yak milk but not characterized in human breast milk (Supplementary Tables S8, S9). Therefore, the metabolite composition of yak and human breast milk was very different.

## 4. Discussion

Metabolites are essential in milk, whereas yak milk metabolites are mainly unexplored. The only report on yak milk was the identification of 21 volatile compounds using GC–MS and GC-O-MS methods (36). In this study, we analyzed the metabolite composition between YC and YMM. Our results show that the metabolism between YC and YMM is very different. According to the method of multi-dimensional analysis and single-dimensional analysis, 109 metabolites with significant differences between YC and YMM were screened, of which 68 were up-regulated, and 41 were down-regulated in YC compared with YMM (Figure 1E). Similarly, 92 metabolites differed between human colostrum (HC) and human mature milk (HMM), with 21 up-regulated and 71 down-regulated metabolites in HC than in HMM (24). Therefore, the number of SDMs between yak colostrum and mature milk (109) is closer in human milk (92). Overall, in human, more metabolites were down-regulated in colostrum compared with mature milk. Conversely, more metabolites were up-regulated in colostrum than mature milk in yak (68 vs. 41). Therefore, we speculate that the metabolite expression trends of yak milk are primarily different from that of human milk during lactation.

Yaks live in the harsh environment of the Qinghai-Tibet Plateau, and hypoxia is an unavoidable major environmental factor in the life of animals on the plateau (37). Arginine, an essential amino acid, is usually deficient in colostrum, but animals are more demanding for rapid postpartum growth (38). More importantly, dietary arginine enhanced hypoxic stress tolerance by reducing hypoxia-upregulated hypoxia-inducible factor (HIF)-1α mRNA expression, inhibiting lipid peroxidation and increasing antioxidant enzyme activity in fish (39). Comparative metabolomic analysis revealed that arginine biosynthesis was the most abundant KEGG pathway among yak-specific metabolites (Figure 6D). Arginine can be synthesized from the glutamine (38). Interestingly, glutamine and glutamate are the second most abundant KEGG pathways among yak-specific metabolites (Figure 6D). Therefore, we speculated that yak milk could enhance tolerance to hypoxic stress by increasing arginine biosynthesis. Hypoxic stress elevates oxidative stress, and the pentose phosphate pathway contributes to increased oxidative stress tolerance in the neonatal brain (40). The pentose phosphate pathway (PPP) is the fifth most abundant KEGG pathway among yak-specific metabolites, not presented in human breast milk (Figures 6C,D). Metabolites in this pathway also showed higher expression levels in YC and YMM (Figure 5). Therefore, yak milk, especially yak colostrum, may also promote tolerance to hypoxic stress by increasing metabolites in the PPP pathway. In humans, glutathione, an amino acid derivate, is vital in reducing oxidative stress, maintaining redox balance, enhancing metabolic detoxification, and regulating the immune system (41). Various age-related chronic diseases are related to poor or insufficient glutathione levels (42, 43). In this study, glutathione was one of the metabolites specific to yak milk but not in human breast milk, and the glutathione concentration in YC was significantly higher than that in YMM (Supplementary Table S8). Therefore, yak milk, especially yak colostrum, may also promote tolerance to hypoxic stress by increasing glutathione levels. Uridine treatment could prevent hypoxia-induced neuro-glial damage to rat brains in neonate (44). Although uridine was commonly expressed in both yak and human milk (Supplementary Table S6), interestingly, the uridine level was higher in yak milk than in human milk (HC: 0.07914, HMM: 0.14402, YC: 2.49364, YMM: 0.26642, Supplementary Table S7). More importantly, the uridine level was decreased in human colostrum than in human breast mature milk. In contrast, yak colostrum had a higher uridine level than yak mature milk (Human vs. yak differ FC: −8.81, Supplementary Table S7). Therefore, yak milk, especially yak colostrum, may also enhance hypoxic stress tolerance by increasing the uridine level. Similarly, glutaric acid-induced oxidative stress in rat brains suggests that it might be a negative metabolite in hypoxic stress tolerance (31). Interestingly, glutaric acid was decreased in yak milk compared to human breast milk (Supplementary Table S7). Thus, yak milk might tolerant the high-altitude stress via decreasing the glutaric acid level. L-isoleucine, L-leucine, and L-valine are essential branched-chain amino acids (BCAAs), and increased BCAAs contributed to the cellular adaptation to hypoxic stress (45). In this study, comparative metabolomics revealed a high L-isoleucine level in yak milk instead of human milk, taken together with L-leucine and L-valine were only presented in yak milk but not characterized in human breast milk (Supplementary Tables S8, S9), we would suggest that BCAAs from yak might also contribute to the cellular hypoxia adaptation. Therefore, yak milk alleviates hypoxic stress via essential amino acid-arginine and branched-chain amino acids. Studying metabolites in yak milk has deepened our understanding of its components and provided a perspective for future research and development of the yak dairy industry.

Although yak milk has diverse metabolites to increase cellular adaptation to hypoxia, some human breast milk metabolites also have their specificity in response to hypoxia (Supplementary Table S9). Citrulline is an amino acid that inhibits hypoxia-induced diseases in newborn pigs and alleviates hypoxia-induced obstructive sleep apnea (OSA) in humans (46, 47). Thus, citrulline is a positive metabolite in hypoxic stress tolerance. Sarcosine is an amino acid that functions in neuroprotection in humans, and it is also a hypoxia-induced metabolite in the extreme hypoxia species Siberian wood frog (48, 49). Therefore, sarcosine might also be a positive metabolite in hypoxic stress tolerance. Creatine, an amino acid derivate, significantly alleviates the hypoxia-induced damage to neuroprotection on a factor called “Complex Attention” (50–52). Thus, Creatine is also a positive metabolite in hypoxic stress tolerance. In this study, citrulline, sarcosine, and creatine were only presented in human breast milk but not in yak milk (Supplementary Tables S8, S9) suggesting that human milk might also function on hypoxic stress tolerance. However, not all amino acids or derivates play a positive function in hypoxic stress tolerance. For example, serine, a non-essential amino acid, catabolism produces the toxic NADH, which is important for aerobic ATP production under the hypoxic stress (53). This study only characterized serine in human breast milk but not yak milk (Supplementary Tables S8, S9), suggesting the complex relationship between human breast milk and hypoxic stress tolerance.

## 5. Conclusion

Yak milk is essential for the survival of Tibetans, and the composition of yak milk during lactation remains unexplored. In this study, 354 metabolites were identified in YC and YMM during lactation. Incredibly, 109 of them were significantly differentially expressed between YC and YMM. Therefore, this study could provide the composition of metabolites of yak milk during lactation, providing a preliminary understanding of yak milk lactation. In addition, comparative metabolomics analysis unveils that yak milk-specific metabolites were enrichment in hypoxic stress-tolerant pathways. Thus, these data also provided helpful information to decipher how the metabolites composition of yak milk contributed to the hypoxic stress tolerance and provide practical information for Yak milk production in the future.

## Data availability statement

The original contributions presented in the study are included in the article/Supplementary material, further inquiries can be directed to the corresponding authors.

## Ethics statement

The studies involving animals were reviewed and approved by the Experimental Animal Welfare and Ethical Review Certificate of Qinghai Academy of Animal Husbandry and Veterinary Sciences (Ethic approval file No. 2023– QHMKY-009). Informed consent was obtained from the owners for the participation of their animals in this study.

## Author contributions

WL and LM: conceptualization. WL, WZ, and XJ: methodology. YZ and XF: software. YZ and ZM: validation. XF: formal analysis. ZM and WL: investigation. YZ: resources. WZ: data curation. WL, WZ, and LM: writing—original draft preparation. WL, LM, WZ, and XJ: writing—review and editing. YZ: supervision. LM: project administration. WL and YZ: funding acquisition. YH and YS: visualization. All authors contributed to the article and approved the submitted version.
