# Supplementary figures and images for: A comparative metabolomics analysis of domestic yak (Bos grunniens) milk with human breast milk
Source: Front Vet Sci. 2023 Sep 29;10:1207950. doi: 10.3389/fvets.2023.1207950 (PMC10570732; doi:10.3389/fvets.2023.1207950)

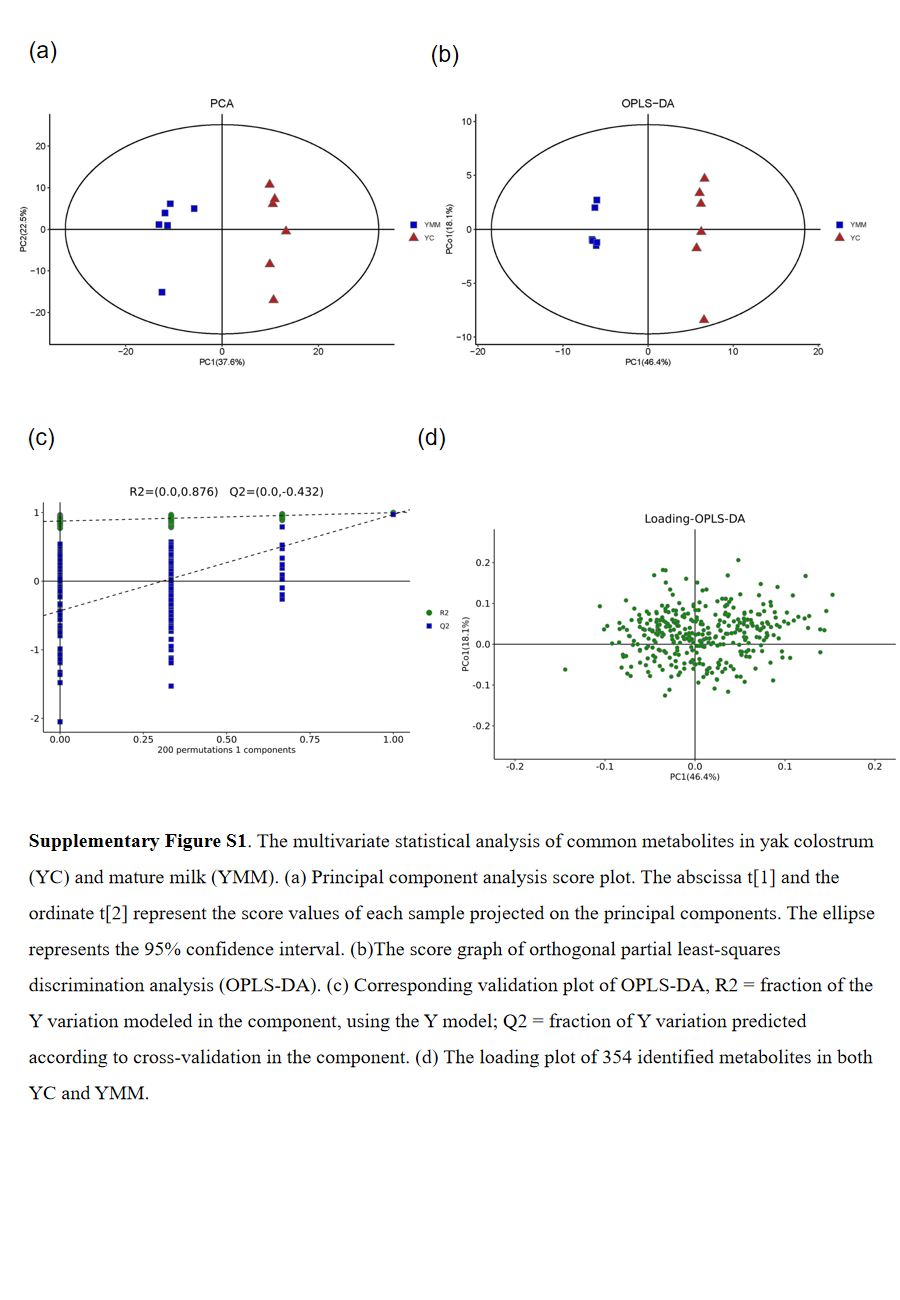

Supplement: Supplementary file 2 [file Image_1.PNG]

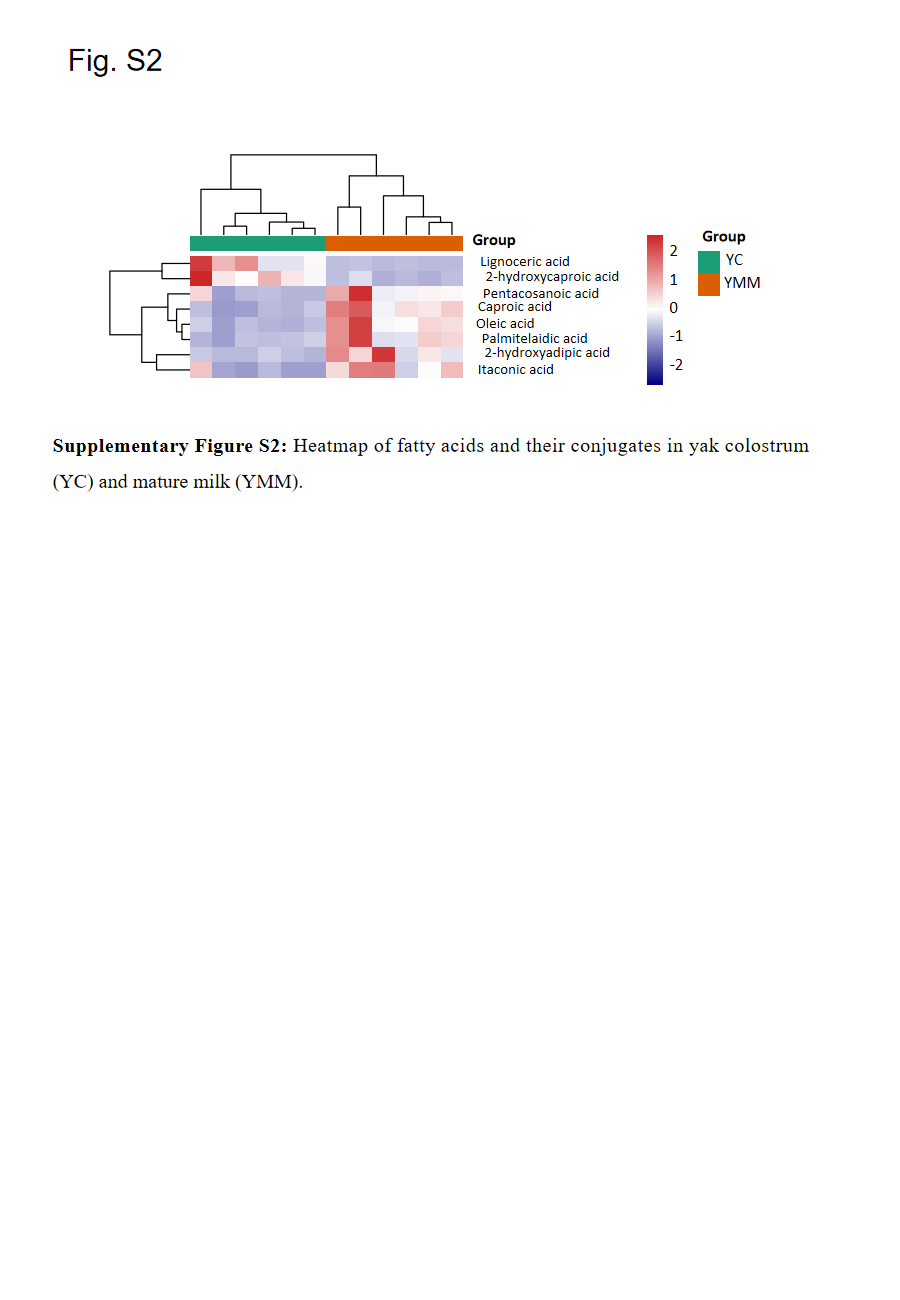

Supplement: Supplementary file 3 [file Image_2.PNG]
